# Breast feeding practices after normal vaginal and caesarean delivery in Gujarat, India

**DOI:** 10.6026/973206300191029

**Published:** 2023-10-31

**Authors:** Ritu Bhati, Gnanadesigan Ekambaram, Mrinalini Gaikwad, Alkesh Vara, B Mahalakshmi, N Sivasubramanian

**Affiliations:** 1 Department of Physiology, GCS Medical College, Hospital and Research Centre, Ahmedabad, Gujarat, India; 2 Department of Physiology, Nootan Medical College & Research Centre, Sankalchand Patel University, Visnagar, Gujarat, India; 3 Department of Anatomy, Medical College & Research Centre, Sankalchand Patel University, Visnagar, Gujarat, India; 4 Department of Paediatric Nursing, Nootan College of Nursing, Sankalchand Patel University, Visnagar, Gujarat - 384315, India

**Keywords:** Breastfeeding, Normal vaginal delivery, Caesarean delivery

## Abstract

Breast feeding is the mainspring of child survival, nutrition, development and maternal health. Early initiation of breastfeeding is an extremely important factor associated with the maintenance of long-term breastfeeding practice. Breastfeeding practices can be influenced by a variety of variables such as parity, mode of delivery; body mass index (BMI), breast or nipple abnormalities and behavioural factors are equally as important. The present study was conducted to analyze Breast Feeding Practices after Normal Delivery and Caesarean Delivery at a Tertiary Care Hospital. This cross sectional study was conducted at tertiary care hospital, by Convenient non-random sampling method, which included two groups; Group A: 100 mothers who delivered vaginally Group B: 100 mothers who gave birth through caesarean section (n=100 each). Participants were asked to complete standardized questionnaire consists of information on socio demographic and breast feeding practice. All anthropometric measurements were taken. A semi-structured questionnaire was used to collect data on maternal socio demographic characteristics, breastfeeding knowledge, practices along with source of information regarding breastfeeding and maternal experience. The study results shows that initiation of breastfeeding is most common in normal vaginal delivery (70%) among total 100 subjects of vaginal delivery category and also common in subjects with planned C-section (49%) of 100 subject"s caesarean delivery category. Association between the modes of delivery and initiation of breastfeeding within an hour was statistically significant (p<0.01). The present study indicates that C-sections are linked to higher breastfeeding challenges, greater resource usage, and shorter nursing duration.

## Background:

Breast feeding is the mainspring of child survival, nutrition, development and maternal health. The Lancet new born survival series found breastfeeding as an intervention that can lower 55 percent to 87 percent of all cause infant mortality and morbidity [1]. Breast milk is best for the baby and the benefits of breastfeeding extend well beyond basic nutrition. In addition to containing all the vitamins and nutrients the baby needs in the first six months of life, breast milk is packed with disease fighting substances that protects baby from illness. The first hour after childbirth is an excellent time to encourage the mother to breastfeed. If mother is successful in breast feeding during first few days of her baby"s life, she is more likely to be successful during the rest of their breastfeeding time [2].The more the baby sucks on the nipple, the more prolactin is released, resulting in increased milk secretion. Exclusive breastfeeding for the first 6 months of life is the recommended way of feeding infants, followed by continued breastfeeding with appropriate complementary foods for up to 2 years or beyond. WHO and the United Nations Children"s Fund (UNICEF) recommend initiation of breastfeeding within the first hour of birth, referred to as "early initiation of breastfeeding." Early initiation of breastfeeding is critical to new born survival and to establish breastfeeding practice over the long term. When breastfeeding is delayed after birth, the consequences can be life-threatening and the longer new born are left waiting, the greater the risk [3]. However, the WHO reported that about 78 million babies, or three in five, are not breastfed within the first hour of life, putting them at higher risk of death or disease and making them less likely to continue breastfeeding [4]. Studies have found that early initiation of breastfeeding is an extremely important factor associated with the maintenance of long-term breastfeeding practice. Studies have reported that breastfeeding reduces neonatal deaths, particularly due to infections [5] such as diarrhoea [6], neonatal sepsis [7] and pneumonia [8]. Breastfeeding also has long-term benefits in the form of improved intelligent quotient, obesity, diabetes, and hypertension [9]. Therefore, all mothers should be supported to initiate breastfeeding as soon as possible after birth, within the 1st h after delivery [10]. Breastfeeding is correlated with a lower risk of certain illnesses in women, such as postpartum bleeding, type 2 diabetes, breast cancer, and ovarian cancer [11]. Breast milk has the most suitable nutrients for an infant"s digestive system and can reduce the risk of gastrointestinal infections, respiratory disease, asthma, and obesity [11]. Breastfeeding rates vary among regions in India [12]. Multiple factors such as socio demographic and obstetric characteristics and cultural beliefs may have an impact on breastfeeding rates [13]. Despite several initiatives taken, exclusive breastfeeding rates remain low.

Breastfeeding practices can be influenced by a variety of variables. Besides known biological factors such as parity, mode of delivery, body mass index (BMI), smoking, breast or nipple abnormalities, surgery, illness, anxiety, and stress, there are behavioural factors that are equally as important [14]. There may also be infant characteristics that contribute to breastfeeding initiation such as gestational age, weight at birth, intrinsic disease, suckling ability, and temperament [14]. Parental attitudes, motivation, and antenatal intentions also play a role in the commitment each individual may have to complete the "birth experience" with breastfeeding. Delivery methods may affect breastfeeding initiation and duration [15]. Multiple studies have found caesarean delivery may hinder breastfeeding initiation [16-17]. Sometimes Breastfeeding initiation becomes a problem after caesarean delivery due to the fact that surgery is associated with inherent risks and difficulties such as longer recovery period than vaginal birth and can cause some complications, including pain, uterine haemorrhage and infections [18-19]. These inherent difficulties and potential complications that can compromise a woman"s ability to breastfeed are of interest. The present study has focused to assess the knowledge, attitude, and practices of breastfeeding and to assess factors associated with breastfeeding practices among postpartum women delivering at a tertiary care hospital in Visnagar. Furthermore, this research aims to determine if caesarean delivery may be a factor impacting early and exclusive breastfeeding in Visnagar to overlook the impact of caesarean section deliveries on timely initiation of breastfeeding practice.

## Methodology:

Type of study: Cross sectional study

Place of Study: Nootan General Hospital/Sankalchand Patel University

Study participants: 200 mothers (100 normal delivery and 100 caesarean mothers)

Study group: Group A: 100 mothers who delivered vaginally Group B: 100 mothers who gave birth through caesarean section

Study duration: 6 months

Sampling method: Convenient non-random sampling

## Inclusion criteria:

18 to 45 years old mothers (who completed first 3 months of post natal life) who delivered in the institute"s maternity center or mothers who are visiting the respective hospital for different purpose and willing to participate in the study were included.

## Exclusion criteria:

Mothers who lost their baby, babies admitted in neonatal intensive care unit prior to starting breastfeeding, and mothers having babies with conditions/malformations where breastfeeding was difficult or contraindicated were excluded from the study.

## Study procedure:

This study is confined only to postnatal mothers. Respondents were made comfortable and clarified about details of the study. Basic clinical examination and vital signs were measured. Participants were asked to complete standardized questionnaire consists of information on socio demographic and breast feeding related information. Confidentiality of responses was maintained. Demographic characteristics were studied. The Present study was approved by Institutional Ethics Committee (IEC) (Approval No: NPDCH/IEC/2022/344). The whole procedure was explained to the subject thoroughly in local language before getting written informed consent from subjects. The assessment was done in accompanied by a female nursing staff or a female attendant. Experiments were done in accordance with revised Helsinki Declaration of 2000.

## Data collection tool:

A semi-structured questionnaire was used to collect data on maternal socio demographic characteristics, breastfeeding knowledge, practices along with source

## Statistical analysis:

Statistical analysis was carried out using SPSS software version 17 for various descriptive statistics. Student t test was used to analyze data between normal delivery and caesarean delivery. P < 0.05 was used as the criterion for statistical significance.

## Results:

**Figure 1:**
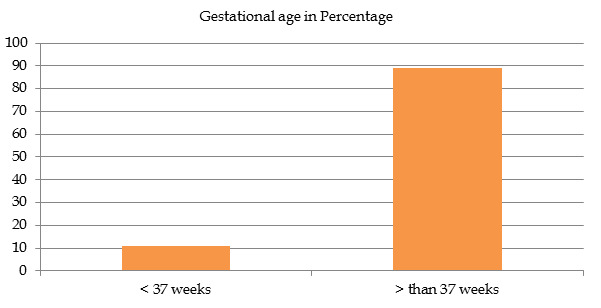
Gestational age in percentage

Table 1 shows demographic characteristics of subjects. Out of 200 mothers 120 (60%) were aged between 18 and 25 years, 46 mothers (23%) were between 26 and 30 years and 34 mothers (17%) were more than 30 yrs.102 (51%) were Primigravida, 98(49%) were multigravida. Majority of participants were belongs to young age, many subjects (91%) had received antenatal care (ANC) while 73% subjects haven"t received counselling regarding breastfeeding during ANC. Table 2 shows the time of breast feeding initiation and infants responses during breast feeding. 33 participants started breastfeeding 24 hours or later, compared to 67% of subjects who started breastfeeding within an hour of caesarean delivery. Majority of the infants (94%) were willing to feed and were able to attach well to the breast (89%). 68% subjects have reported that breast feeding was continued up to 3 months of post natal life, had skin to skin contact (89%). Table 3 displays the mother"s experiences with starting and continuing nursing within 24 hours of a caesarean section. The majority of respondents (70%) said they believed it was simple and comfortable to breastfeed. Other details were given in table 3.75% women had awareness regarding importance of colostrum. 80% women had initiated only breastfeeding (Table 4). Table 5 showed modes of delivery and initiation of breastfeeding within an hour. Association between the modes of delivery and initiation of breastfeeding within an hour was statistically significant (p<0.01). It also shows initiation of breastfeeding is most common in normal vaginal delivery (70%) among total 100 subjects of vaginal delivery category and also common in subjects with planned C-section (49%) of 100 subject"s caesarean delivery category. Figure 1 showed 89% women had completed 37 weeks of gestational period.

## Discussion:

The present study shows only 27% of women received counselling regarding breastfeeding during antenatal visit. These findings were supported by research conducted by Romola *et al.* [20] who found that only 16.5% of moms received breastfeeding advice during prenatal appointments. Thus, this study emphasises the significance of bringing up nursing during antenatal visits. The present study showed the importance of first milk (colostrum"s) is known to many mothers. In the present study, the initiation of breastfeeding is most common in normal vaginal delivery. It is in agreement with Johar *et al.* [21]. In contrast to the Malaysian National Health and Morbidity Survey (NHMS) of 2016, this indicated that only 49% of moms who gave birth via caesarean section started nursing within an hour of giving birth, our proportion of early breastfeeding initiation within an hour significantly greater [22]. In a study of Puerto Rican women, a lower percentage of breastfeeding was initiated (61.5%), while a significantly higher percentage (97.5%) of breastfeeding was initiated by Canadian women during their stay in the hospital following caesarean delivery [23].

According to UNICEF, 42% of new born worldwide started nursing during the first hour of their lives in 2018. Although our study"s (70%) rate of early breastfeeding initiation among women undergone vaginal delivery within the first hour of birth is significantly higher than the global rate, there is still room for improvement. According to a systematic review and meta-analysis on the relationship between delayed breastfeeding initiation and infant survival, among infants who were exclusively breastfed during the neonatal period, those who started breastfeeding more than 24 hours after birth had an 85% higher risk of neonatal mortality than those who started breastfeeding less than 24 hours after birth [24]. Because breastfeeding can save lives and has long-term benefits, there is a need to step up efforts to boost the rate of breastfeeding initiation within the first hour of life [25]. Skin-to-skin contact between infants and their mothers, giving the baby the time to naturally begin his or her first sucking response, has been linked to an increase in the initiation of breastfeeding. About majority of the respondents in the current study had skin-to-skin contact with their new born [26] (Table 2). It is in agreement with Johar *et al.* [21]. In a study conducted in 2017 by Boyd, a similar finding was made: 74% of post-caesarean moms initiated skin-to-skin contact in the operating room or in the post-anesthesia care unit. According to a 2016 systematic analysis by Moore *et al.* women who engaged in skin-to-skin contact after a caesarean birth were more likely to successfully breastfeed their children for 1 to 4 months following the birth [27]. Increase of skin-to-skin contact among caesarean women is necessary since research demonstrates that it improves rates of exclusive breastfeeding, lengthens breastfeeding, and enhances the likelihood that babies will be nursed [2].

In our study, women who underwent an emergency C-section were more likely to have tried nursing once before failing, to be unable to do so for the first 24 hours after giving birth, and to still be unable to do so after leaving the hospital. It is in agreement with Amy J. Hobbs *et al.* [28]. Similar findings were made by Zanardo *et al.* (2010), who noted that women who underwent emergency C-sections were more likely to have been unable to nurse their infants at either the time of birth or when they were discharged [29]. It has been suggested that early postpartum breastfeeding difficulties and early discontinuation may be related to the mother and foetal stress response associated with delivery issues, particularly those related to C-sections [31]. Lactogenesis may be affected by both emergency C-sections, planned C-sections, although the idea of emergency may elicit a prolonged or greater maternal stress response. It could also be brought on by a woman's eating habits and physical and mental endurance. According to a research by Evans *et al* [31] in comparison to women who gave birth vaginally, women who had C-sections transferred much less breast milk in the first five days following delivery, Similar findings were made by Scott and Binns (2007), who discovered that mothers who gave birth via C-section had considerably greater rates of delayed lactation than mothers who gave birth vaginally [32]. The hormonal system that promotes lactogenesis is thought to be disturbed after C-section birth, either as a result of maternal stress or a decrease in oxytocin release, and this could have been the reason behind reduces milk production [33]. Although prior breastfeeding experiences were not examined in this study, it is possible that special consideration may be given to women who have had trouble breastfeeding in the past in order to encourage future successful breastfeeding experiences. From the perinatal phase through the postpartum period, more focused interventions for women expecting C-section deliveries may promote breastfeeding initiation and maintenance. As a result of our study's finding that women who undergo emergency C-sections are more likely to struggle with breast feeding and need additional lactation support, these mothers could receive focused instruction on breast feeding techniques and encouragement as soon as possible after giving birth in order to give them the right kind of anticipatory advice to lessen difficulties. The postpartum home visit gives an excellent chance to offer further breastfeeding exams and assistance to these women, as women who gave birth by emergency C-section were more likely to not have nursed successfully before leaving the hospital.

## Conclusion:

Data indicates that C-sections are linked to higher breastfeeding challenges, greater resource usage, and shorter nursing duration. In order to guarantee early success with nursing, it is also advised that additional supportive care be made available to lactating women who had emergency C-section throughout the immediate to early postpartum period. Hence, it is crucial that health professionals emphasise the significance of early breastfeeding initiation to women who intend to have caesarean deliveries starting in the antenatal stage. The present study helps to understand the prevalence of breast feeding practices and to create awareness about breast feeding among postnatal mothers after normal and caesarean delivery.

## Figures and Tables

**Table 1:** Demographic characteristics of subjects

**Characteristics**		**Number**	**Percentage (%)**
Age	18-25	120	60
	26-30	46	23
	>30	34	17
Education	Secondary	18	9
	Higher secondary	88	44
	Graduate and Post graduate	94	47
Occupation			
	Employed	20	10
	Homemaker	180	90
Received ANC	Yes	182	91
	No	18	9
Have you received counselling regarding breastfeeding during ANC	Yes	54	27
	No	146	73
Socioeconomic status	Upper Class	10	5
	Middle Class	50	25
	Lower Class	140	70
Residency	Rural	160	80
	Urban	40	20
Family type	Nuclear	186	93
	Joint family	14	7
Tobacco use	Yes	4	2
	No	196	90
	Occasionally	6	3
	Passive smoker (Cigarette / Tobacco)	194	5
Parity status	Primipara	102	51
	Multiparous	98	49

**Table 2:** Time of breast feeding initiation and infants responses during breast feeding initiation among women undergone vaginal & caesarean delivery

**Variable**	**Response**	**Vaginal delivery (%)**	**Caesarean delivery (%)**
Time of breastfeeding	Within 1 h	75	67
	>1 h to 23 h	22	30
	24 h and more	3	3
Had skin-to-skin contact	Yes	90	89
	No	10	11
Breast feeding duration	Yes	75	68
Continued up to 3 months of post natal life	No	25	32
Infant's responses during breastfeeding initiation	Yes	96	94
Infant willing to feed	No	2	3
	Not sure	2	3
Infant looks sleepy	Yes	15	25
	No	75	68
	Not sure	10	7
Infant able to attach well to the breast	Yes	93	89
	No	4	6
	Not sure	3	5

**Table 3:** Maternal experience toward breastfeeding initiation and practice among post natal mothers (C Section mothers)

**Variable**	**Often**	**Sometimes**	**Seldom**	**Never**
Feels easy and comfortable	70	19	7	4
Feels confident that breast milk is adequate for baby	68	18	11	3
Difficulty to move due to pain	55	35	8	2
Feeling tired	29	24	20	27
Had headache or dizziness	4	19	17	60
Pain at surgical site	41	35	22	2
Perceived no milk	8	20	16	56
Had cracked /inverted nipple	3	12	7	78
Breast pain as baby suckle	6	18	20	54

**Table 4:** Awareness about breast feeding

**Variable**	**Response**	**N**	**Frequency %**
Colostrum should be discarded	Yes	30	15
	No	150	75
	No idea	20	10
Breast feeding in first 6 months	Only breast feeding	160	80
	Breast feeding with other food	40	20

**Table 5:** Association between the modes of delivery and initiation of breastfeeding within an hour

**ode of delivery**	**Initiation of breast feeding in the 1st hour of the delivery**		**Total**	**P value**
	**Yes (%)**	**No (%)**		
Normal delivery (spontaneous)	23 (77)	7 (28)	30	
Normal delivery (induced)	49 (70)	21 (30)	70	
Caesarean (Emergency C section)	11 (35)	22 (71)	31	
Caesarean (Planned C section)	34 (49)	35 (51)	69	P<0.01
